# Consumption of a low glycaemic index diet in late life extends lifespan of Balb/c mice with differential effects on DNA damage

**DOI:** 10.1186/2046-2395-2-4

**Published:** 2013-03-01

**Authors:** Scott A Nankervis, Jenee M Mitchell, Fadi J Charchar, Maree A McGlynn, Paul A Lewandowski

**Affiliations:** 1 School of Health Sciences, University of Ballarat, Ballarat, Australia; 2 School of Medicine, Deakin University, Geelong, Vic 3217, Australia

## Abstract

**Background:**

Caloric restriction is known to extend the lifespan of all organisms in which it has been tested. Consequently, current research is investigating the role of various foods to improve health and lifespan. The role of various diets has received less attention however, and in some cases may have more capacity to improve health and longevity than specific foods alone. We examined the benefits to longevity of a low glycaemic index (GI) diet in aged Balb/c mice and examined markers of oxidative stress and subsequent effects on telomere dynamics.

**Results:**

In an aged population of mice, a low GI diet extended average lifespan by 12%, improved glucose tolerance and had impressive effects on amelioration of oxidative damage to DNA in white blood cells. Telomere length in quadriceps muscle showed no improvement in the dieted group, nor was telomerase reactivated.

**Conclusion:**

The beneficial effects of a low GI diet are evident from the current study and although the impact to telomere dynamics late in life is minimal, we expect that earlier intervention with a low GI diet would provide significant improvement in health and longevity with associated effects to telomere homeostasis.

## Background

Caloric restriction (CR) has positive effects on both mean and maximum lifespan [1-4], and since the original report the lifespan and health-span increasing capabilities of CR have been documented in a range of animals ranging from yeast to flies and mammals [2,5]. These studies indicate that CR-induced increases in animal longevity are accompanied by decreased morbidity from a range of age-related diseases such as cardiac and cardiovascular disease, autoimmune disease, cancer, diabetes and renal, respiratory and neural diseases [2,6,7]. Furthermore, CR has also been implicated in stress resistance responses [7,8]. The most potent example of a form of CR is dietary methionine restriction which has been documented to increase lifespan by 45% in F344 mice [8,9], while protein restriction yields an average 20% lifespan increase in mice and rats [2,3,9]. It is now clearly evident that diet plays a crucial role in health and longevity, and unraveling the active interacting components of diet and ageing could have positive ramifications worldwide.

Telomere length decrease has emerged as a potential marker of biological ageing [2,10] and the overarching idea suggests that telomeres protect the ends of chromosomes from replicative loss; the unavoidable shortening of chromosomes that occurs with successive rounds of division eventually leads to chromosomal damage, at which point the cell enters a state of irreversible senescence [10]. The combination of dynamic telomeres with the lifespan-increasing capabilities of CR and dietary restriction (DR), have made telomeres an important target for longevity research. In dissecting various contributors to the success of CR and DR, researchers have become aware of the ability to influence telomere length maintenance and fluctuation with the provision and/or restriction of certain foods. In a large study of individuals on a range of dietary patterns, Nettleton *et al*. [7,11] did not identify significant impacts to leukocyte telomere length in any of a range of foods studied, except for processed meat which showed a negative correlation with telomere length with increasing intake. In a health study using only female participants, dietary fibre was identified as having a positive association with leukocyte telomere length [8,12], while chronic DR (defined here as restricted eating due to chronic preoccupation with weight) was found to shorten leukocyte telomere length in a female cohort [8,9,13]. In men placed on CR diets, O’Callaghan *et al*. [10,14] observed midrectal mucosa telomere length gain with increasing weight loss over a 12-month period; the more weight lost, the greater the increase in telomere length. Studies on the effect of lifelong CR on rhesus monkeys have reported an increase in longevity for CR animals [11,15], however this is not associated with CR-induced alterations to skeletal muscle telomere length [1,12] and may be an artifact of husbandry and study design rather than CR itself [5,13]. Work by Wang *et al*. [6] in which mice were placed on short-term (3-month) CR programs revealed improvements to telomere length maintenance without increases in telomerase activity in the small intestine, and reduced frequency of senescent intestinal enterocytes and liver hepatocytes. Evidence to date certainly indicates beneficial effects of CR/DR programs to both longevity and telomere length.

It is apparent that one of the contributing factors to improved longevity and telomere length on a CR or DR program that decreases caloric intake is that of reduced oxidative stress through decreases in steady-state oxidant levels; oxidative damage is believed to be a key factor causing senescence [7,15]. A number of studies have shown a link between CR and decreased production of reactive oxygen species (ROS) [3,4,6,7,16,17], and oxidative damage to DNA (oxidised bases, abasic sites and single- and double-strand breaks) increases with ageing in mammalian tissues [3,4,18]. Additionally, DR has been shown to decrease cumulative markers of oxidative stress in intestine and liver of mice and contributes to a decrease in cellular senescence [2,6]. The link between oxidative stress and telomere shortening specifically is now well established. Von Zglinicki *et al*. [19] reported reductions in telomere length in human fibroblasts exposed to oxidative stress, and that this loss results in telomere-driven replicative senescence as a stress response. Telomere shortening was also observed after oxidative stress induction in a variety of tissues in mice [7,20]. The oxidative damage to telomeres has been localised specifically to the GGG triplet of the telomeric repeat [8,21,22] and it has been made apparent that telomeres are particularly susceptible to damage caused by oxidative stress. Components of the protective shelterin complex of telomeres (such as TRF2) prevent access by DNA damage repair (DDR) mechanisms and this induces persistent DDR, markers of which are evident in telomeres that are not critically short in terminally differentiated cells [23,24]. The susceptibility to damage and the inability to repair induces cellular senescence, and this can occur independently of telomere length [23,24]. It is now apparent that although telomere length is a critical factor in ageing, exogenous forms of stress acting specifically on telomeres are also a crucial in this regard and can induce senescence without telomere shortening.

CR (and DR) is clearly linked to beneficial improvements in health and longevity in animals and humans [2,9]. Telomeres prevent cellular senescence occurring as a consequence of DNA damage such as occurs via oxidative stress, and dietary factors are known to prevent ROS accumulation. Due to the links among telomeres, oxidative stress and dietary intake, the impact of diet on telomere biology and ageing is an important area only now beginning to receive attention. This is certainly apparent in the associations between glycaemic index (GI) and oxidative stress. Chronic hyperglycaemia is correlated with increased production of free radicals and depletion of antioxidants in patients with type 2 diabetes [10,25], and positive associations with markers of oxidative stress were observed with elevated glycated haemoglobin and blood glucose concentration in patients with well-controlled type 2 diabetes [11,26]. In the present study, we examined the impact of a low GI diet on ageing mice, examining markers of health and longevity and their consequent impacts on aspects of telomere biology.

## Results

### Glycaemic index of the test diets

**Figure 1:**
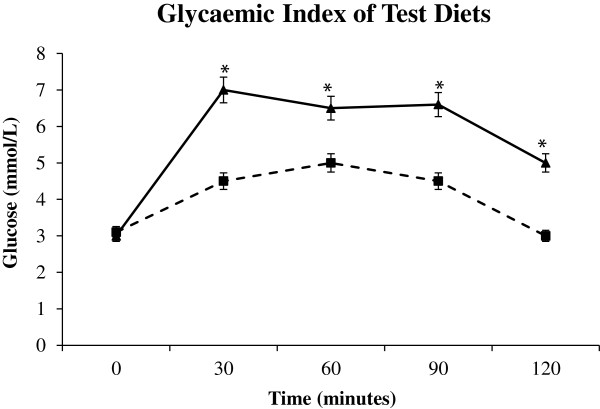
**Glycaemic index of the experimental diets.** Plasma glucose after ingestion of 500 mg of experimental diets. Asterixes indicate significant difference in blood glucose concentration at each measured interval (*P* <0.05). Error bars show SEM. Solid line with triangular markers (-▲ -) denotes control group, perforated line with square markers (-- ■ --) denotes low GI group.

After the training period, eight animals in each group consumed the entire test meals from each diet. Differences for glucose at single time points are shown in Figure 1. The low GI diet resulted in significantly lower blood glucose at all time points except for baseline (*P* <0.05).

### Lifespan

**Figure 2:**
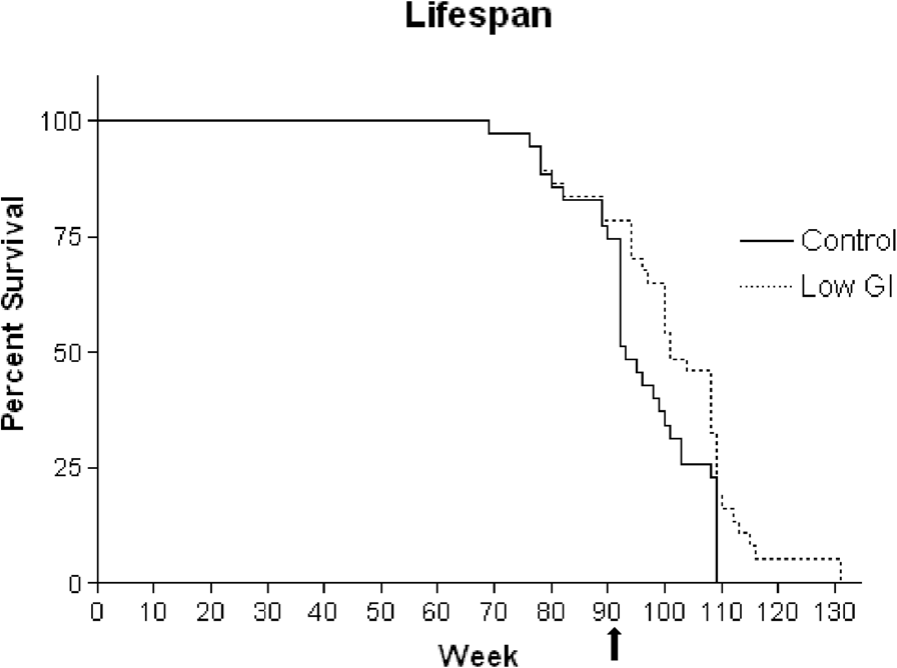
**Lifespan of control and low GI mice.** The low GI intervention commenced at week 90 (20 months old, arrow). Solid line (-) denotes control group, perforated line (- -) denotes low GI diet group.

A Kaplan-Meier plot of mouse survival (Figure 2) indicates the survival rates of control and low GI mice until termination of the study. The low GI diet intervention started at 90 weeks (20 months old). There was a significant difference in average lifespan between control (95 weeks) and low GI (106.7 weeks) groups (*P* = 0.00003), that represented an increase of approximately 12% in lifespan for animals on a low GI diet. Approximately 25% of the original population died before 90 weeks, indicative of the aged status of this population prior to intervention and showing that a low GI diet significantly extended the lifespan of animals that had already entered old age.

### Body weight and body weight to muscle ratios

**Figure 3:**
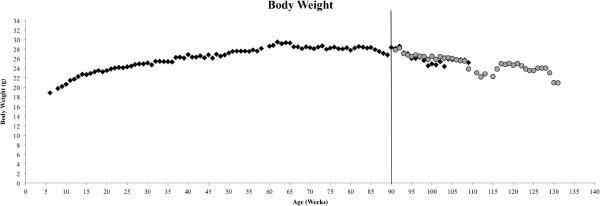
**Average body weight measurements per week for all animals measured until mortality.** Vertical line at week 90 indicates beginning of low GI intervention for the experimental group. Black diamonds (♦) denote control group, gray circles (•) denote low GI group.

Mouse body weight was recorded as the average body weight of all animals each week over the course of the study and is shown in Figure 3. Expectedly, body weight increased to maturity and declined with ageing; body weight of the low GI mice continued to decline into extreme old age until the weight of the last remaining animals nears values seen for the 6-month-old mice. Average body weight increased steadily until maximum average body weight was attained at week 62 and reached 29.5 g ± 0.25 SEM. Average body weight was maintained consistently until approximately week 85 and began to decline as mortality increased (Figure 2). Low GI intervention was begun on a subset of animals at week 90 and separate average weights calculated for this group beginning at week 91. Average body weight increased at this time point due to the deaths of some of the comparatively underweight individuals (data not shown) that increased the average, but this smoothed out from week 93 onwards until week 109 where this phenomenon was seen again. Week 110 marks the death of the final control animals and body weight becomes consistent again amongst the low GI population until the conclusion of the study with the death of the last remaining animals.

### Food and water consumption

**Figure 4:**
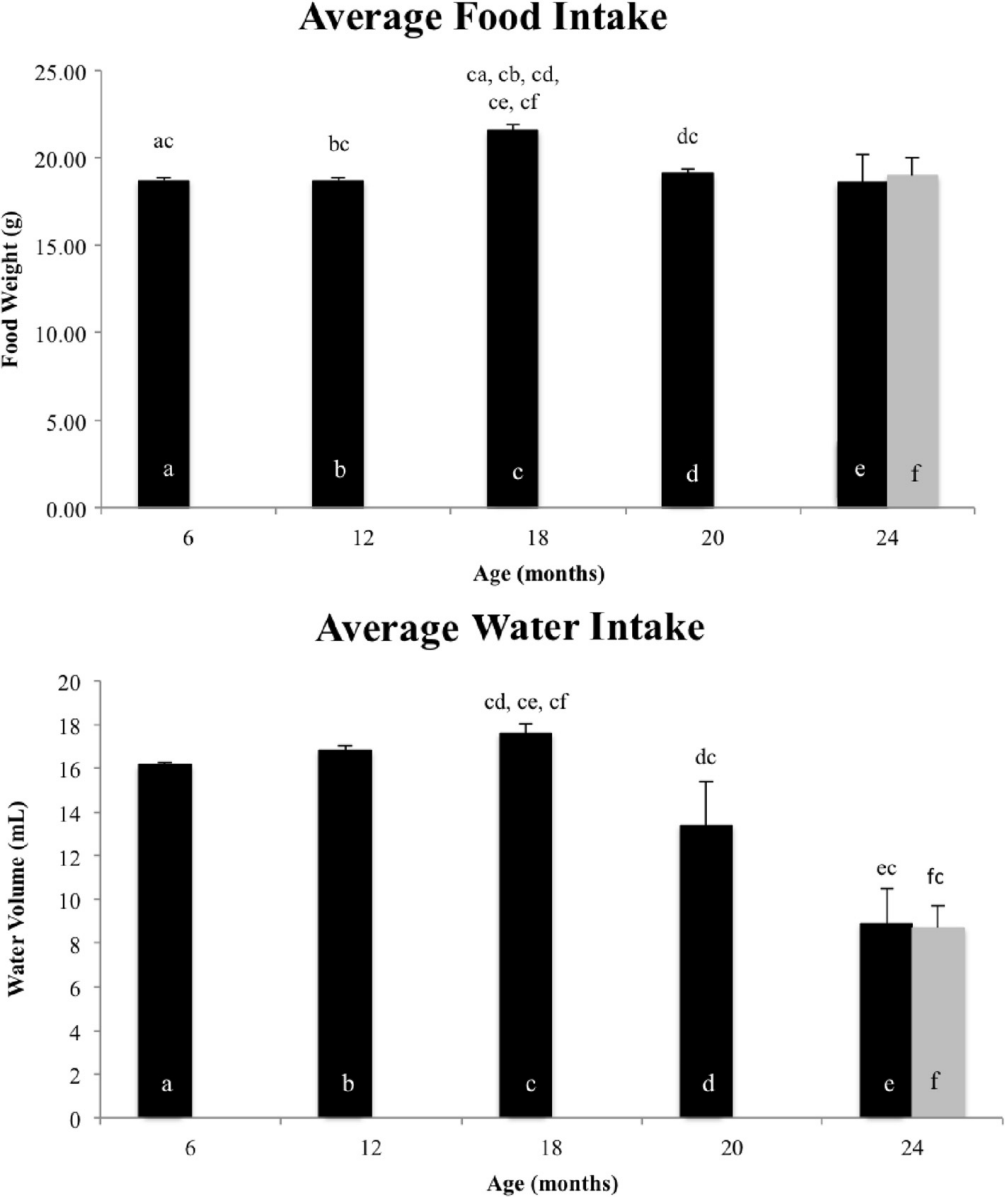
**Average food and water intake.** (**A**) Average food intake for all animals (*n* = 8 for each group); a significant increase in consumption was observed in 18-month-old animals; black bars indicate control groups, gray bars indicate low GI group; (**B**) Average water intake for all animals (*n* = 8 for each group); significant decreases in consumption were observed with age from 18 months old onwards. For both (**A**) and (**B**), each age group has been designated a to f, and significant differences for each age group compared to other age groups are indicated above the SEM, for example, cd indicates that group c (18 months old) is significantly different to group d (20 months old); black bars indicate control groups, gray bars indicate low GI group.

The average quantity of food consumed increased at 18 months of age, and then declined and remained steady (Figure 4A). There was no difference in food quantity consumed between control and low GI animals at 24 months of age. Average water intake rose steadily with age until 18 months of age, and then decreased dramatically with increasing age afterwards (Figure 4B). Significant decreases in intake were observed between 18- and 20-month-old animals and 18- and 24-month-old animals, however there was no observed difference in intake between 24-month-old control and low GI populations.

### Intraperitoneal glucose tolerance test

**Figure 5:**
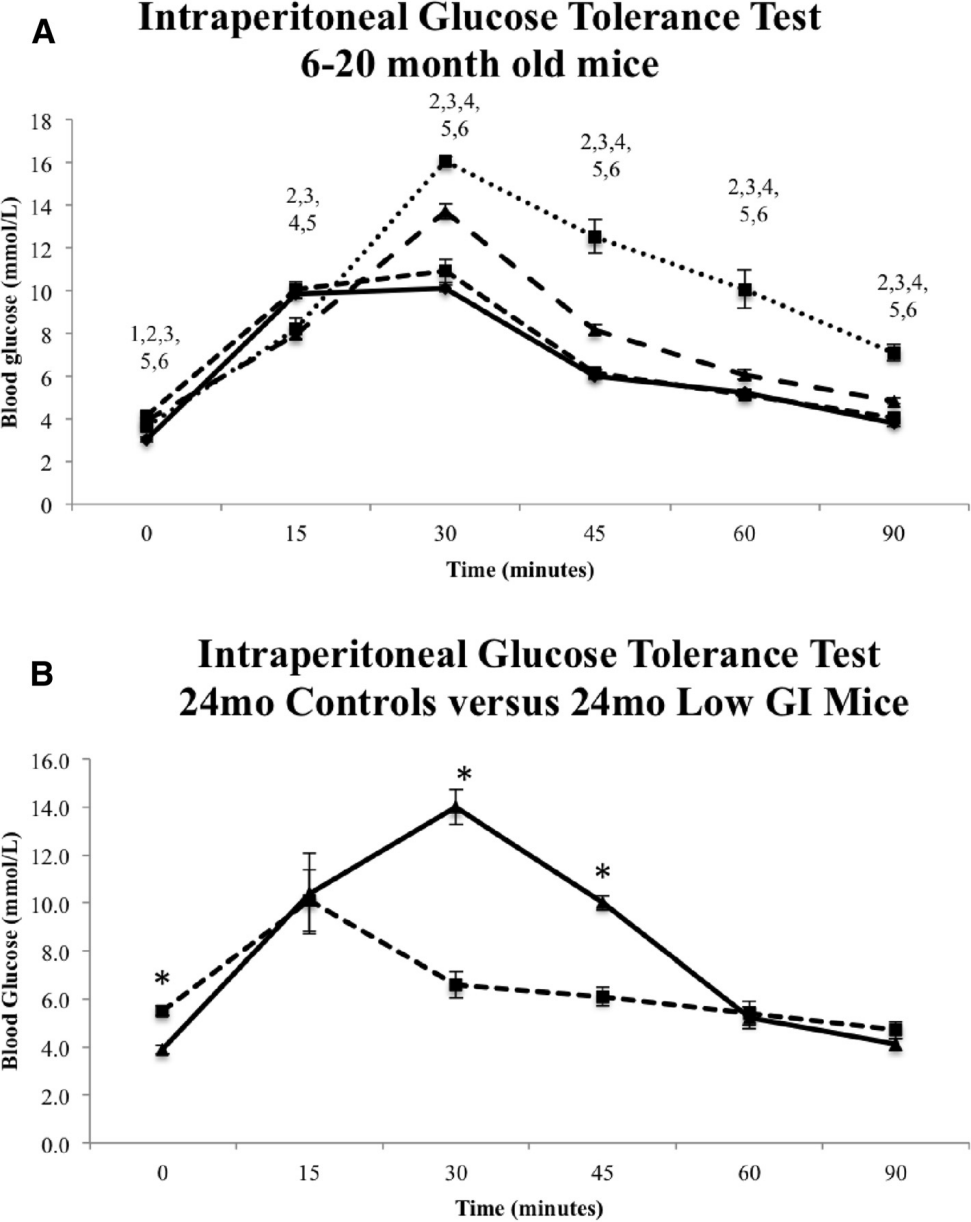
**Blood glucose tolerance.** (**A**) Intraperitoneal glucose tolerance measured in mmol/L over a period of 90 min in 6- to 20-month-old control mice (*n* = 16 each group); numbers above data points at each time interval indicate significant differences according to the following scheme: 1, 6mo *vs.* 12mo; 2, 6mo *vs.* 18mo; 3, 6mo *vs.* 20mo; 4, 12mo *vs.* 18mo; 5, 12mo *vs.* 20mo; 6, 18mo *vs.* 20mo. Error bars show SEM. Solid line () denotes 6mo group, while perforated lines from longest to smallest denote 12 (- -), 18 (- - -) and 20mo (^…^) controls respectively. (**B**) Intraperitoneal glucose tolerance measured in mmol/L over a period of 90 min in 24-month-old control and low GI diet mice (*n* = 8 each group). Asterixes indicate significant difference in blood glucose concentration at each measured interval (*P* <0.05). Error bars show SEM. Solid line with triangular markers (-▲ -) denotes control group, perforated line with square markers (- -■--) denotes low GI group.

The glucose tolerance of mice was measured to assess the physiological impact of a low GI diet intervention in old age (Figure 5). Aside from some differences among age groups in resting blood glucose concentration prior to glucose injection (t = 0), from 30 min onwards we observed consistent significantly elevated blood glucose concentration with age with the exception of younger mice (6 and 12 months old) in which there was no difference in glucose clearance rate after injection.

In the control or low GI groups at 24 months old we observed either increases or no difference in blood glucose concentration in the older animals compared to other age groups (Figure 5B). Interestingly, for the most part there appears to be consistent reductions in blood glucose concentration in 24-month-old low GI mice in comparison to other age groups, while the control counterparts consistently show increased blood glucose concentration in comparison to all other age groups. Furthermore, while the 24-month-old control and low GI mice had similar (though significantly different) resting and end-point blood glucose concentrations, there were significant differences at 30 (*P* <0.0001) and 45 (*P* = <0.0001) min post injection which reflect a much greater capability of the low GI mice to tolerate blood glucose fluctuation.

**Figure 6:**
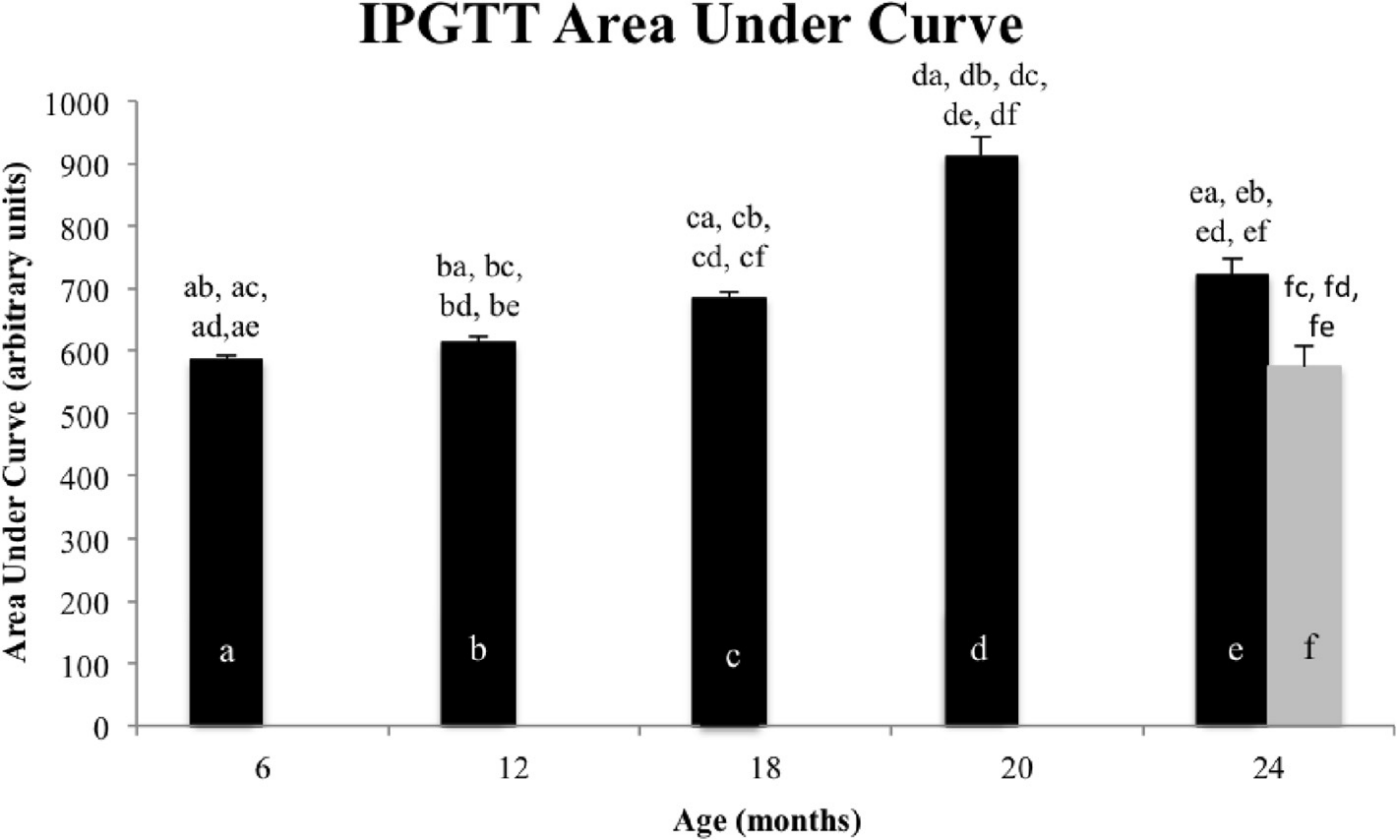
**Area under curve measured for IPGTT in all mice age groups.** Each bar is designated a to f, and all corresponding significant differences are indicated above each bar (*P* <0.05), for example ab indicates that group A (6 months old) is significantly different to group B (12 months old); black bars indicate control groups, gray bars indicate low GI group, (*n* = 16 for 6- to 20-month-old groups, *n* = 8 for 24-month-old groups).

We calculated the area under the curve (AUC) for each age group (Figure 6). It is apparent from the data that there is a relationship between age and glucose tolerance with a steady significant increase in AUC up to the 20-month-old time point, reflecting age-related glucose intolerance. Surprisingly, the 24-month-old control mice show a similar AUC value to 18-month-old animals (no significant difference; *P* = 0.2), while the 24-month-old low GI group has an AUC value similar to mice between 6 and 12 months old (no significant difference to 6 months old, *P* = 0.75, or 12 months old, *P* = 0.3). The difference between 24-month-old control and low GI groups is significant with a decrease in AUC for the low GI group, reflecting greater glucose tolerance (*P* = 0.004).

### Oxidative stress

**Figure 7:**
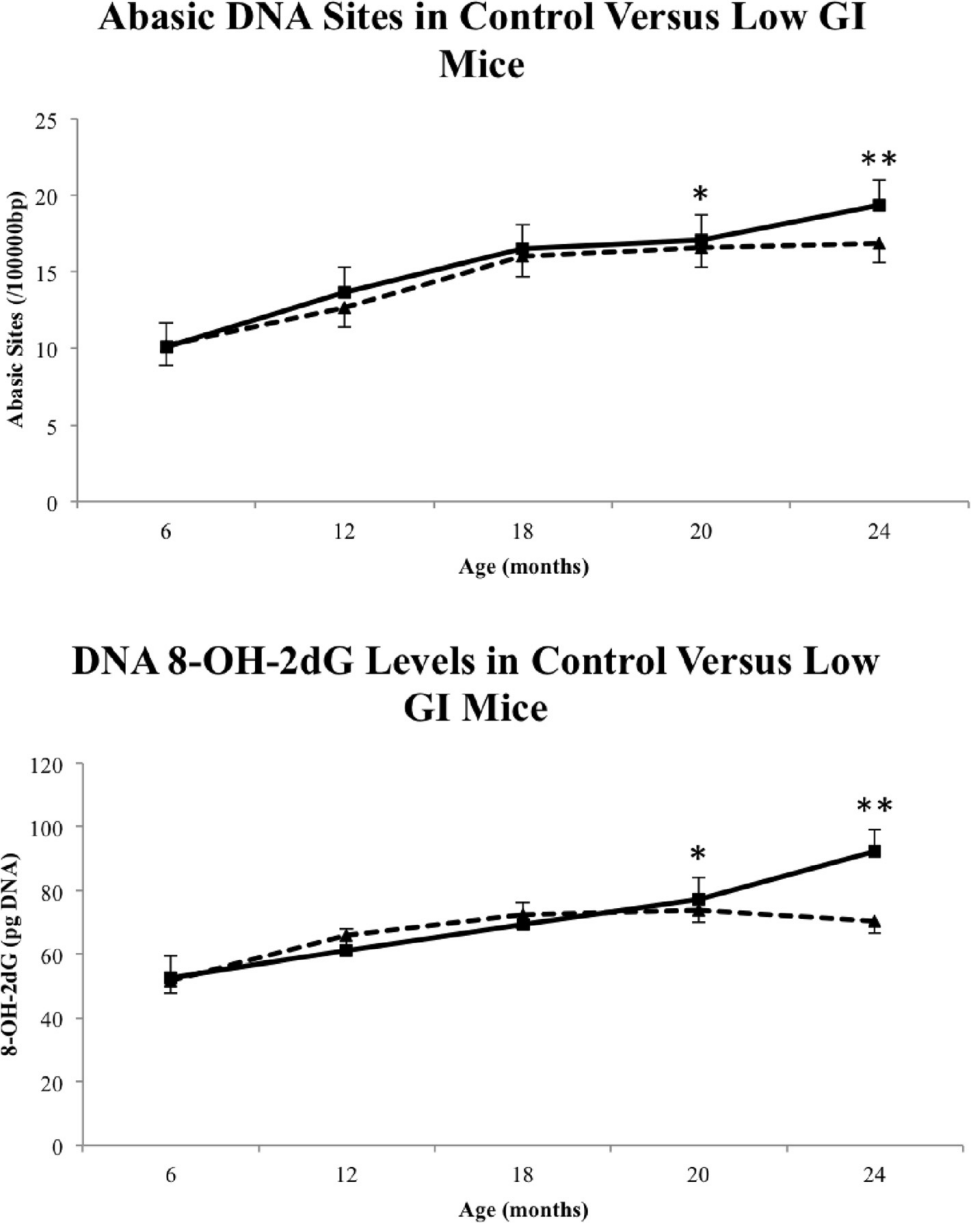
**Markers of oxidative stress in control *versus *low GI mice.** (**A**) Abasic DNA sites were identified in both control mice and the low GI group mice at each time interval and data shown are number of abasic sites per 100,000 base pairs (bp) (*n* = 16 for 6- to 20-month-old animals, *n* = 8 for 24-month-old animals); * indicates significant difference between 20-month-old control *versus* 24-month-old control, ** indicates significant difference between 24-month-old control *versus* 24-month low GI (*P* <0.05); solid line () denotes control, perforated line (- - -) denotes low GI. (**B**) Levels of 8-OH-2dG formation in DNA in both control mice and low GI mice at each time interval, data shown are pg/mL of 8-OH-2dG formed (*n* = 16 for 6- to 20-month-old animals, *n* = 8 for 24-month-old animals); * indicates significant difference between 20-month-old control *versus* 24-month-old control, ** indicates significant difference between 24-month-old control *versus* 24-month low GI (*P* <0.05); solid line () denotes control, perforated line (- - -) denotes low GI.

Aged mice have significantly reduced markers of oxidative stress after intervention with a low GI diet (Figure 7), as measured by frequency of abasic DNA sites and accumulation of 8-OH-2dG in genomic DNA isolated from WBCs. We observed significant increases in these two markers of oxidative stress between 20 and 24 months old in the control population (abasic *P* = 0.007, 8-OH-2dG *P* = 0.01), but this increase was ameliorated in the low GI intervention group to the point that abasic frequency and 8-OH-2dG levels remained steady from 20 months old onward. There was also a significant difference between control and low GI groups at 24 months old (abasic *P* = 0.002, 8-OH-2dG *P* = 0.001), with greater levels of oxidative stress as indicated by both markers observed in the control group.

### Telomere length

**Figure 8:**
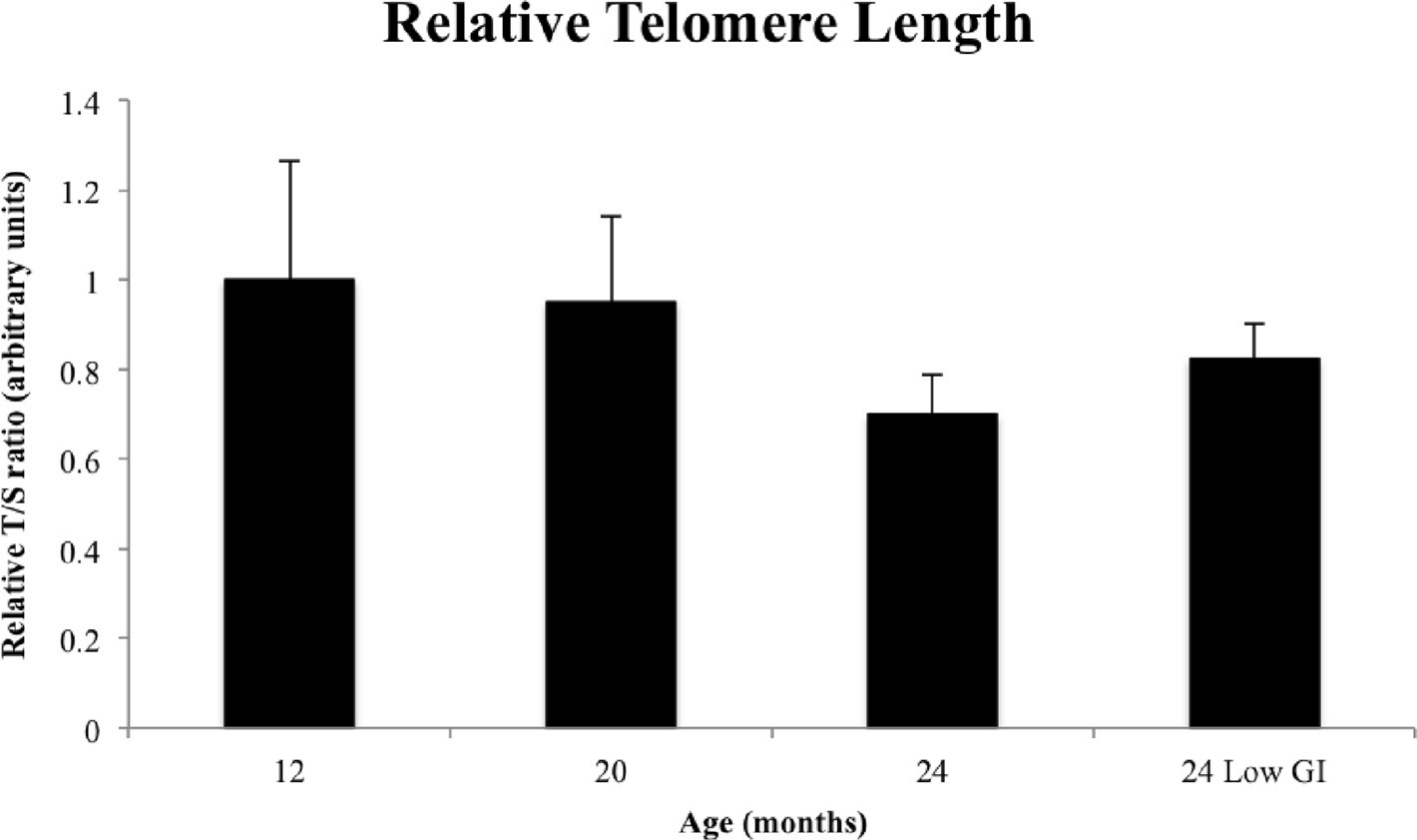
**Relative telomere lengths of mice.** Data for 12-month-old mice were normalised to 1, and the telomere lengths for other age groups were calculated relative to the 12-month-old group. No significant differences were observed between any of the groups, (*n* = 6, 8, 7 and 7 for the 12, 20, 24 and low GI groups, respectively).

Since dietary regime is known to influence telomere length homeostasis [1,6,12-15], we measured average quadriceps muscle telomere length of all mice up to 20 months old, and in both the control and low GI diet groups (Figure 8). The variability of the data at the 12-month-old and 20-month-old groups is evident for these time points, however this variability is decreased by 24 months. There was no significant difference found in quadriceps muscle irrespective of the reference point used, however there does appear to be a trend in declining telomere length with age that is possibly reversed with a low GI diet.

### mTERT gene expression

As a marker of telomere dynamics additional to length change, we measured the gene expression of mTERT (rate limiting component of telomerase) in mouse quadriceps. The expression of mTERT was not detectable until 24 months of age, and was inconsistently detected (data not shown).

## Discussion

Lifespan extension via CR is well documented in the literature [14,27], and there is evidence implicating DR, specifically protein restriction, with lifespan extension as well [8,9,15]. Often, studies examining CR or DR also find changes in telomere length, commonly used as a marker of biological ageing [1,6,12-15]. We have observed lifespan extension of aged mice placed on a low GI diet, yet did not find any associated changes to telomere length.

The effect of a CR diet on longevity is wholly dependent on the point in the life of the organism at which the regime is started [5,28]. Bearing this in mind, the present study is limited in reporting the extent to which a low GI diet can positively affect mouse longevity; however at even such a late stage in life, we were able to significantly extend the average lifespan of aged mice through a low GI diet alone by 12%. In studies examining the effect of lifetime CR, mice fed a slightly restricted calorie diet showed a 13% increase in lifespan compared to *ad libitum* controls [6,29], while CR has been used in 19-month-old mice to extend mean time to death by 42%, and increase both mean and maximal lifespan [7,30]; 34% increase in lifespan [3,6,7,16,17] and a negative correlation observed between caloric intake and average lifespan [31] has also been reported. So while the evidence supporting the life-extending capability of CR is apparent, it appears from the present study that a low GI diet can be similarly potent. The average lifespan of control mice was 95 weeks indicating that the 90-week (20 months) intervention point is after approximately 95% of life lived which is well in excess of the calculations (for humans) provided by Speakman and Hambly [28]. This suggests that there would effectively be no beneficial impact of a CR diet at this point in the animals’ life, which agrees with Lipman *et al*. [32]. It is of interest then that our use of a low GI diet has produced a 12% increase in lifespan from late-life (90 weeks) which is later than predictions [28] and other studies [30] have produced with CR. This suggests that the use of a low GI diet from earlier in life has the potential to elicit even greater lifespan effects and warrants further investigation.

Lifetime body weight fluctuation in mice typically increases over the first 12 months, and then plateaus [29,33]. We report similar increases in body weight for the first 12 months (approximately 60 weeks old), which plateaus until week 85 and begins to steadily decline with age, unlike the mice or rats in the aforementioned studies but somewhat similar to Masoro *et al*. [34]. The calculated quadriceps muscle to body weight ratio (Figure 3) confirm the rapid growth from 6 to 12 months old with a sharp decline at 18 months that plateaued for the remainder of the study, and this agrees well with the literature [3,29,33]. This decline may indicate that the mice in our present study behaved physiologically as other rodents do, but without weight maintenance (based on continuous weight decline with age: Figure 2); this could be explained by fat composition in the diet (Table 1), however this is not reported in the Weindruch [29] or Alemaan [33] studies. Typically, CR results in reduced body fat (reviewed in [27]) which would produce a divergence in body weights of *ad libitum versus* CR animals over time; since our intervention was late in life, there is unlikely to have been sufficient time to observe noticeable differences in body weight in the present study. Once this factor is removed, the decline in weight with age is probably attributable to decreased muscle mass, a phenomenon known as sarcopenia which is typical in ageing animals [35]; however even this is seen to be reduced in rats [36] and monkeys [37] on a CR diet.

**Table 1:** Diet composition

**Ingredients**	**Low GI**^**a**^	**Control**^**b**^
Digestible energy (MJ/kg)	13.5	14.0
Protein (%)	19.7	20.0
Fat (%)	5.0	4.8
Crude fibre (%)	6.4	4.8
Valine (%)	1.40	0.87
Leucine (%)	0.88	1.40
Isoleucine (%)	1.40	0.80
Threonine (%)	0.80	0.70
Methionine (%)	0.23	0.30
Cystine (%)	0.31	0.30
Lysine (%)	1.00	0.90
Phenylalanine (%)	0.90	0.90
Tyrosine (%)	0.7	0.50
Tryptophan (%)	0.20	0.20
Total monounsaturated fats (%)	1.47	2.00
Total polyunsaturated fats (%)	1.72	1.77
Total saturated fats (%)	0.45	0.74
Mineral mix S10001 (g/kg)	35	35
Vitamin mix V10001 (g/kg)	10	10
Glycaemic index (*vs.* glucose)	27	70

^a^A fixed formula ration including barley, lupins, canola oil, safflower (high linoleic) oil, dicalcium phosphate, magnesium oxide, calcium carbonate, salt, sulphur, fish meal, soya bean meal, vitamin premix and mineral premix. Diet was primarily based on barley and designed to reduce GI using published data on raw materials used.

^b^A fixed formula ration including wheat, barley, lupins, soya meal, fish-meal, mixed vegetable oils, canola oil, salt, calcium carbonate, dicalcium phosphate, magnesium oxide, vitamin premix and mineral premix. Diet was primarily based on wheat with GI determined using published data on raw materials used.

**Figure 9:**
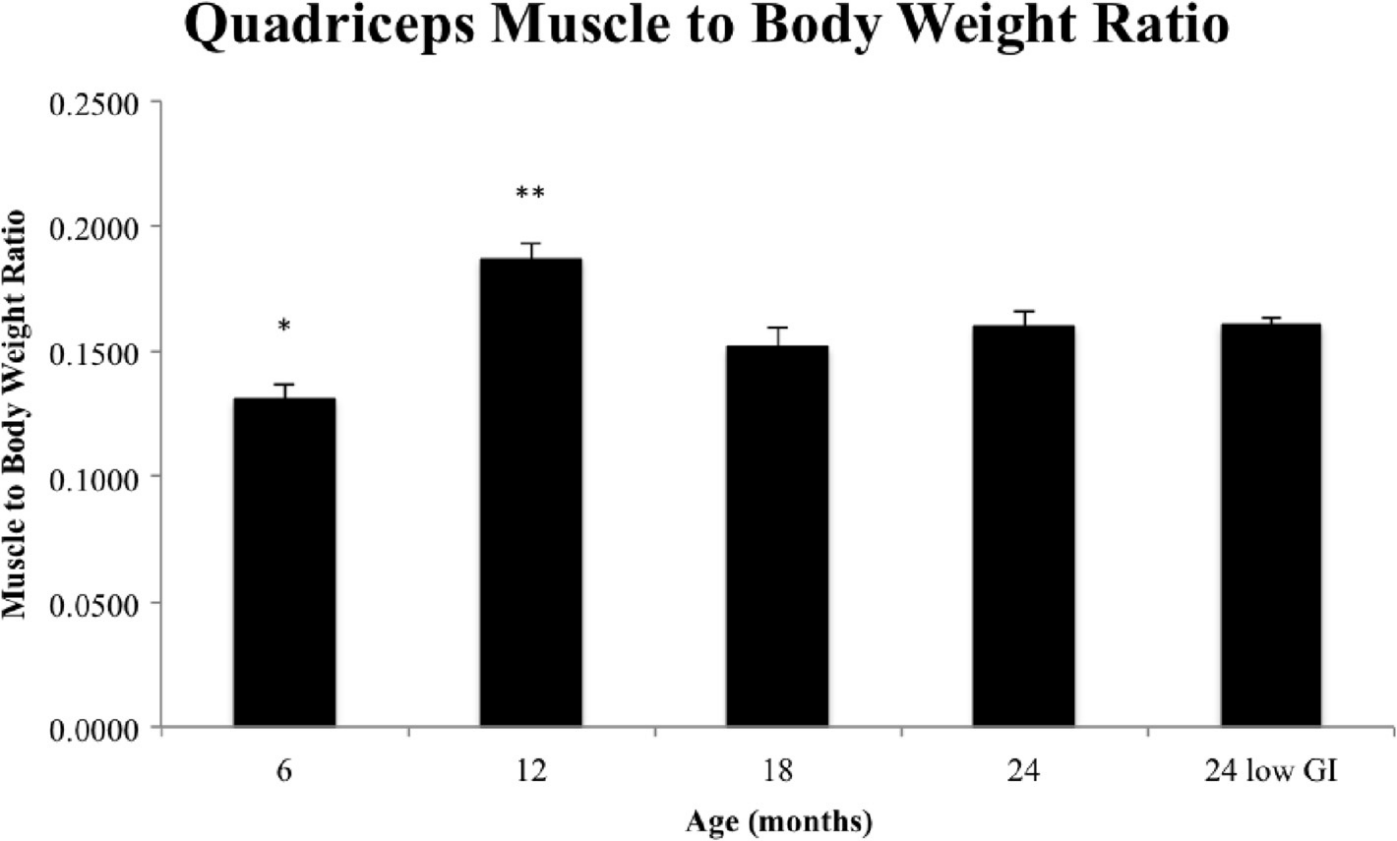
**Muscle to body weight ratios for ageing mice.** Average quadriceps muscle to body weight ratio (*n* = 8 for each age group). One-way ANOVA indicated significance among the groups (*P* <0.001). An asterisk indicates a significant difference between the 6-month-old group and every other group except 18 months old (*P* <0.05); two asterisks indicate a significant difference between the 12-month-old group and every other group (*P* <0.05).

There is a clear relationship between food intake and body weight (Figures 2 and 9A). Food intake continuously increased up to 18 months of age, whereafter it decreased, and the change in body weight over time reflects this food intake with a decline with increasing age after 18 months. Our observation of similar food intake between control and low GI groups is in contrast to the increases observed in low GI animals [38]. When comparing water intake in the low GI and control groups there was no difference in consumption however we observed a downward trend in water consumption with age. Elderly humans are known to have decreased thirst sensitivity [39,40], and this phenomenon seems a possibility in our mice since in old age mouse body weight approximated those of the 6-month-old measurements and food intake did not change, yet decreased water intake was observed. This however could also be the result of activity levels, which we did not record, typically being higher in younger animals (and requiring more water) and declining with age.

Decreased glucose tolerance is a hallmark of both diabetes mellitus [41] and ageing [42]. Reflecting this as a consequence of ageing, we observed a steady decline in glucose tolerance with age in mice until 20 months old, after which tolerance began to increase slightly (and significantly) in the 24-month-old controls and then returning to tolerance levels observed in 18-month-old animals, while those on a low GI diet had their glucose tolerance improve to levels observed at 6 months of age (Figure 5). The response of the low GI animals in the present study is likely similar to the concomitant increases in insulin sensitivity that have been observed in rats undergoing CR [43] and on low GI diets as evidenced by smaller area under curve for blood glucose and plasma insulin after oral glucose challenge [38]. Therefore, as glucose intake is reduced (as in low GI diet), the animal becomes more sensitive to the effects of insulin and rapidly removes circulating glucose. It is difficult to reconcile the improvement in glucose tolerance in the 24-month-old controls, particularly when food consumption did not decrease. Based on the steady, sharp decline in water intake from 18 months onwards it is possible that thirst sensitivity declined with age and the subsequent hypovolemia may have increased circulating insulin concentration which improved glucose tolerance transiently; the further significant increase in glucose tolerance seen in low GI animals could be the result of both decreased thirst sensitivity and improved insulin sensitivity.

The link between age-related mortality and increases in oxidative stress have been documented well and in particular the effect of a CR regime in mammals is shown to reduce oxidative stress and increase lifespan [7,16,44]. It has been suggested that oxidative stress is linked to mortality with a negative correlation between specific metabolic function (greater in smaller animals) and life span [45] - increased oxygen consumption to support increased metabolism exposes the organism to higher quantities of oxidants [46,47]. We have demonstrated a potential link between low GI diet and lifespan with oxidative stress as an intermediate (as measured by number of abasic sites and formation of 8-OH-2dG) (Figure 6) in the present study. Furthermore while the current study focused on oxidative to DNA it is conceivable that a low GI diet may reduce other biomarkers of oxidative stress, including glutathione, malondialdehyde and protein carbonyls. The effect of oxidative stress appears to be particularly pronounced in telomeric *versus* non-telomeric sequence, specifically the GGG triplet, and this focus of oxidative damage may be important in age-related telomere shortening [21,48], or early senescence without shortening [23,24]. Associations between telomere length and ageing were first made apparent by Cawthon *et al*. [49] with shorter telomeres contributing to greater all-cause mortality. Following this, other groups reported findings of shortened telomeres contributing to early mortality and/or cellular senescence [50-52]. Subsequently, the causes of telomere shortening, in addition to cellular division, have become a point of interest and include genetics, oxidative stress, inflammation and psychological/life stress [10]. The capacity for muscle telomere length to reflect environmental damage to myocytes has been indicated by Ahmad *et al*. [53] in a type-2 diabetes setting and is preferentially used over white blood cell leukocyte telomere length. In the context of our present study, placement on a low GI diet impacted glucose tolerance and therefore glucose metabolism; therefore we reasoned that measurement of muscle telomere length reported by Ahmad *et al*. [53] was an appropriate experimental approach. Furthermore, recent studies are revealing that skeletal muscle has far greater capacity for telomere regulatory mechanisms than previously realised [54-58]. While we observed a downward trend in telomere length in mice overtime, we did not observe any significant differences over the course of this trend. It is clear from Figure 7 that there was great inter-individual variation in telomere length in mice up to 20 months old, and this is in fact supported in the literature [59]. The inter-individual variability is also known to decline with age [60], an effect we have seen in the present study. Whether the apparent, slight non-significant increase in telomere length in the 24-month-old low GI group is a trend could be determined with earlier intervention with a low GI diet. It is possible that in combination with the reduction in markers of oxidative stress in low GI animals that continued telomere length measurement in this group might indicate telomere length maintenance and/or slowed progression of shortening while control animals continue to decline. However, with the most recent advances in understanding the effects of oxidative stress-induced damage to telomeres we cannot rule out the possibility that telomere length is not the important factor here, but could instead be early senescence caused by irreparable DNA damage [23,24] which is attenuated by reduced oxidative stress in the low GI group.

To tease out changes to telomere dynamics with intervention using a low GI diet, we measured the gene expression of mTERT, the subunit of telomerase responsible for telomere lengthening. Telomerase activity is known to increase with intensive lifestyle intervention (which included diet) [61], however the majority of human cells have no detectable telomerase activity [62]. Telomerase is transcriptionally regulated [62] and so TERT is a good proxy for the detection of resumption of telomere lengthening in adult cells. The lack of consistent mTERT gene expression in mouse quadriceps indicates that in this tissue a low GI diet failed to reactivate telomerase gene expression, and therefore appreciable telomere lengthening and/or length homeostasis did not occur via this mechanism. Combined with the observed lack of telomere lengthening in GI-dieted animals, a lack of mTERT detection suggests that any role a low GI diet may have at the telomere level in lifespan extension may be through length maintenance instead.

## Conclusions

The present study has identified the potential benefit of a low GI diet to increase lifespan. Compellingly, we were able to extend mouse lifespan from a very late intervention point suggesting that a long-term regime could be a very powerful agent for longer, healthier life. The most direct indication of low GI impact was in markers of oxidative stress that were significantly decreased at 24 months of age in the low GI diet group. We did not observe significant alterations to telomere length but did observe a slow decline with age, nor did we observe changes in TRF2 gene expression indicating that protective mechanisms were not altered at this level over the course of the study. The relevance of the findings to humans would still needs to be tested. However it is possible to speculate that consumption of a low GI diet late in life still has beneficial health effects and it may extend lifespan, although the effect may not be as dramatic as an intervention earlier in life. The extension of life may not represent a significant number of years lived but rather an improved quality of life or healthspan.

## Availability of supporting data

The data set supporting the results of this article is included within the article.

## Methods

### Animal model of ageing and low GI diet supplementation

All animal procedures carried out in this study were approved by the Deakin University animal welfare committee (A37/2007). Four-week-old female Balb/c mice (*n*=70) were assigned to five groups: 6, 12, 18, 24 months and a group that would be allowed to achieve their natural lifespan. Mice were housed in groups of up to eight animals per box and maintained under controlled environmental conditions; 12-h light/dark cycle, 21 ± 2°C, 30% humidity, in conventional cages with *ad libitum* access to standard chow (Table 1, Specialty Feeds) and water up to 20 months of age. After 20 months the mice in the 24-month group and mice that were still alive in the life span group were allocated to receive *ad libitum* access to a low GI chow and water (*n*=32, Table 1, Specialty Feeds) or continued with *ad libitum* access to standard chow and water (n=32) for the remainder of the study. Animal body weights, food intake and water consumption were measured once a week, while the health of the animals was monitored daily. Twenty months of age was estimated to be equivalent to a human age of 75 years based on an average lifespan of 21.1 months in mice being equivalent to a human lifespan of 85 years. Animal life span was determined based on mice being found dead in their cage or being euthanised for animal welfare purposes. At 6 (*n=*16), 12 (*n=*18), 18 (*n=*12) and 24 (*n=*12) months mice were anaesthetised via intra-peritoneal injection with lethabarb (50 mg/kg), then blood and skeletal muscle tissues were collected and immediately snap frozen in liquid nitrogen and stored at -80°C for later use.

### Glycaemic index of the test diets

In addition to the main experiments, postprandial glucose responses to the low GI and control diets were tested in a separate group of 12-week-old female Balb/c mice (*n*=8 for each diet). In order to achieve comparable food intake with both diets, eight mice per group were trained for 5 days. Animals consumed a small portion of standard chow, by introducing a 1 g pellet into the cage of individually housed mice at 08:00, following an overnight fast. After 10 min the leftover pellets were removed. During the training period, mice were given free access to standard chow from 11:00 to 20:00. On day 5, animals received 500 mg of experimental diets, low GI or control. Blood samples from the tail vein (0 min) for measurement of blood glucose were drawn in the overnight-fasted state. Further blood samples were drawn only from mice that consumed the whole portion of the test meals within 5 min (30, 60, 90 and 120 min) and blood glucose analyses via a glucometer (Optium Xceed, Abbott Diabetes Care).

### Blood processing

Blood was collected from the tail vein into EDTA coated tubes and samples were centrifuged at 1,200 × *g* for 10 min at 4°C. The plasma was then removed and stored at -80°C. The buffy coat, containing white blood cells (WBC), was also removed and stored at -80°C until analysis of DNA damage.

### Intraperitoneal glucose tolerance test

Similar to the glycaemic index test, after fasting for 6 h, animals were anaesthetised with an intraperitoneal injection of pentobarbitone sodium (100 mg/kg) and a silastic catheter filled with heparinised saline (20 U/mL) was inserted into the left carotid artery. Mice also underwent a tracheotomy to aid with breathing. A bolus of glucose was injected into the intraperitoneal cavity and 200 μL of blood was sampled at 0, 15, 30, 45, 60, and 90 min for blood glucose analyses via a glucometer (Optium Xceed, Abbott Diabetes Care).

### Oxidative damage to DNA

Oxidative damage to DNA was assessed by measuring the number of abasic sites and 8-hydroxy-2-deoxy guanosine (8-OH-2dG) in WBCs. Genomic DNA was extracted from the buffy coat using a DNA isolation kit (Promega). Abasic sites were determined in genomic DNA from WBC using a DNA damage quantification kit (Dojindo Molecular Technologies), according to the manufacturer’s instructions.

An ELISA kit was used to measure the genomic DNA oxidation byproduct 8-OH-2dG (StressMarq Biosciences). Each sample was then diluted so that 50 ug of DNA was used in the 8-OH-2dG assay. The competitive immunoassay involves the binding of free 8-OH-2dG to an antibody coated 96-well plate. The assay and sample concentration of 8-OH-2dG were carried out as per the manufacturer’s instructions.

### Nucleic acid extraction and preparation for qPCR

Genomic DNA was extracted from frozen quadriceps muscle using the Bioline ISOLATE Genomic DNA Mini Kit (Bioline, Australia). DNA and total RNA concentration was determined spectrophotometrically at 260 nm, and complimentary DNA (cDNA) was synthesised from 1 μg RNA using the QIAGEN QuantiTect® Reverse Transcription Kit following the manufacturer’s instructions (QIAGEN, Australia).

### Telomere length measurement

To measure telomere length in quadriceps muscle, we used the qPCR method published by Cawthon *et al*. [63] with primer sequences modified for telomere measurement in mice by Callicott and Womack [64]. Mouse telomere primer sequences were as follows: CGG TTT GTT TGG GTT TGG GTT TGG GTT TGG GTT TGG GTT and GGC TTG CCT TAC CCT TAC CCT TAC CCT TAC CCT TAC CCT (5^′^ to 3^′^ forward and reverse respectively). These primers have been designed to minimise the formation of primer dimers and are documented to perform similarly to terminal restriction fragment length analysis in both mice and humans [63,64]. The highly conserved acidic ribosomal phosphoprotein PO (36B4) gene was chosen as a single-copy reference gene; these sequences were as follows: ACT GGT CTA GGA CCC GAG AAG and TCA ATG GTG CCT CTG GAG ATT (5^′^ to 3^′^ forward and reverse respectively). PCR reactions were prepared using SensiMix SYBR No-ROX master mix (Bioline, Australia) as previously described [63] and cycled on a Mastercycler® ep realplex (Eppendorf, Australia) thermocycler. Fluorescence emission data were captured and telomere length was analysed as described previously [63] and reported as relative telomere length.

### Taqman qPCR assays

Gene expression assays in quadriceps muscle for mTERT and control 18 s rRNA were prepared using a TaqMan Gene Expression Master Mix (Applied Biosystems, USA) specific for each gene following the manufacturer’s instructions. Reactions were cycled on a Mastercycler® ep realplex (Eppendorf, Australia) thermocycler, with cycling conditions as per the manufacturer’s instructions (Applied Biosystems, USA). To compensate for variations in input RNA amounts and efficiency of reverse transcription, 18 s rRNA was quantified and all results were normalised to these values. Fluorescence emission data were captured and mRNA levels were analysed using the cycle threshold value (CT) [65].

### Statistical analyses

Statistical analysis was performed using SPSS statistical package (version 15.0). Results were expressed as mean ± SEM. Differences were determined by one-way ANOVA with Tukey HSD as *post hoc*, or by a Mann-Whitney *U* test where appropriate and results were considered statistically significant if *P* values ≤0.05.

## Competing interest

The authors declare that they have no competing interest.

## Authors’ contributions

SAN carried out the analysis and interpretation of data and drafted the manuscript. JMM carried out laboratory procedures relating to telomere length and qPCR. FJC contributed to the interpretation of data and revised the manuscript. MAM carried out all experiments and methods involving animals and revised the manuscript. PAL conceived and designed the study, carried out all other laboratory analysis decribed, contributed to the interpretation of data and revised the manuscript. All authors read and approved the final manuscript.
